# Dental Anomalies in 1p36 Deletion Syndrome: A Case Report

**DOI:** 10.7759/cureus.96719

**Published:** 2025-11-12

**Authors:** Faris A Alotaibi, Mohammed K Alotaibi, Tarfa N Moharib

**Affiliations:** 1 Department of Pediatric Dentistry, King Saud Medical City, Riyadh, SAU; 2 Department of Pediatric Dentistry, Security Forces Hospital, Riyadh, SAU

**Keywords:** 1p36 deletion syndrome, dental anamolies, infraocclusion, mesiodens, permanent canines agenesis

## Abstract

This report describes a nine-year, five-month-old Saudi girl with 1p36 deletion syndrome (1p36DS) referred for dental evaluation due to esthetic concerns. Clinical and radiographic assessment revealed multiple carious lesions, poor oral hygiene, dens invaginatus in maxillary incisors, infraocclusion of a primary molar, and, notably, agenesis of all primary and permanent canines except for one retained mandibular primary canine. Additionally, two supernumerary maxillary incisors (mesiodens) were identified, causing severe rotation of adjacent teeth, a combination of dental anomalies not previously documented in 1p36DS.

A comprehensive treatment plan was executed, encompassing preventive measures, restorative care, surgical extraction of mesiodens, and interceptive orthodontic alignment using a 2×4 fixed appliance and transpalatal arch. These interventions led to improved esthetics, dental function, and psychosocial confidence.

The management of 1p36DS patients requires a multidisciplinary approach due to the conjunction of craniofacial and systemic anomalies. Pediatric dentists play a crucial role in early detection, preventive care, and timely interventions, all of which contribute to enhanced function and quality of life. Routine panoramic imaging and vigilance for atypical dental patterns are critical for guiding genetic referrals and comprehensive care.

This case broadens the known phenotypic spectrum of 1p36DS by documenting a novel dental presentation involving concurrent agenesis of both primary and permanent canines with supernumerary maxillary incisors. Early recognition of such anomalies enables timely diagnosis, personalized management, and multidisciplinary collaboration, emphasizing the vital role of pediatric dentists in the holistic care of patients with rare chromosomal disorders.

## Introduction

Chromosome 1p36 deletion syndrome (1p36DS) is the most common terminal chromosomal deletion in humans, with a prevalence estimated at 1 in 5,000 to 1 in 10,000 live births [1,2]. It accounts for approximately 0.5-1.2% of all cases of syndromic intellectual disability [3,4]. Clinically, 1p36DS presents with a broad spectrum of manifestations, including developmental delay, intellectual disability, hypotonia, seizures, craniofacial dysmorphism, congenital heart defects, cardiomyopathy, hearing impairment, visual deficits, and short stature [5,6]. Typical facial features include straight eyebrows, deep-set eyes, midface hypoplasia, a broad nasal bridge, and a pointed chin [4,7].

The syndrome is caused by heterozygous deletions of varying size on the short arm of chromosome 1, leading to haploinsufficiency in several critical genes [4,8]. There is marked phenotypic variability among individuals, likely due to differences in deletion size and location, contiguous gene effects, and possible parental origin influences [3,8].

Although the systemic features of 1p36DS have been extensively described, the dental phenotype is less well characterized. Recent reports have noted anomalies such as agenesis, delayed eruption, and abnormal tooth morphology [2]. Dental agenesis, in particular, has been suggested as a potential additional feature of the syndrome, highlighting the need for comprehensive dental evaluation in affected individuals [2]. Furthermore, perioperative dental management may be complicated by existing craniofacial and neuromuscular anomalies [1].

This case report details a unique dental phenotype in a patient with 1p36DS, specifically the presence of supernumerary maxillary incisors (mesiodens) alongside the agenesis of all primary and permanent canines. To the authors' knowledge, this combination has not been previously reported.

The family of the patient have consented, and a written consent form was obtained from them.

## Case presentation

A nine-year, five-month-old Saudi girl from a rural area was referred in February 2024 to the Pediatric Dentistry Clinic at a tertiary hospital in Riyadh, Saudi Arabia, due to concerns about the esthetics of her anterior teeth. Her father reported that the patient experienced psychosocial distress, including repeated bullying at school, because of her dental appearance.

**Figure 1:**
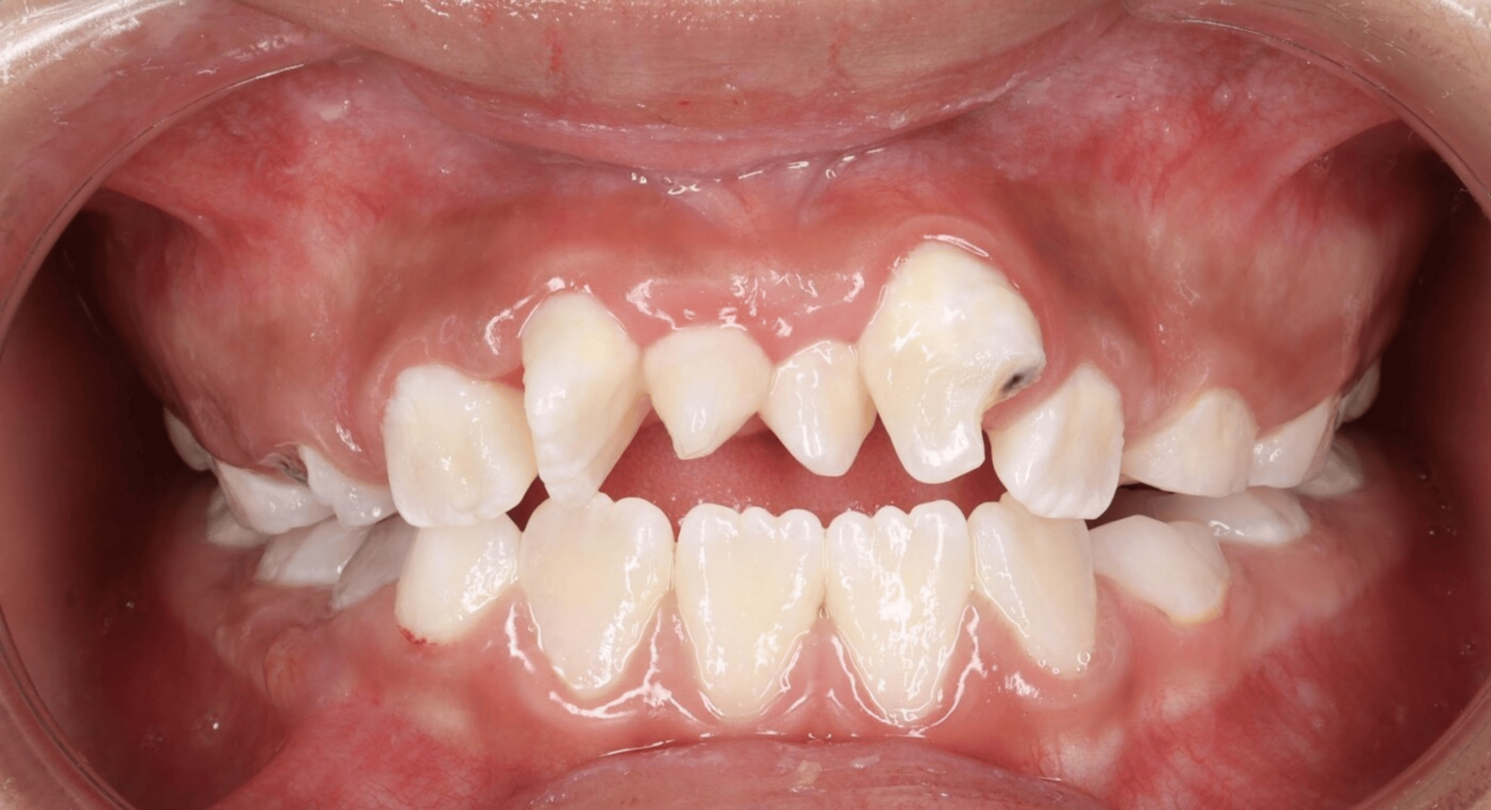
Frontal view of the patient

**Figure 2:**
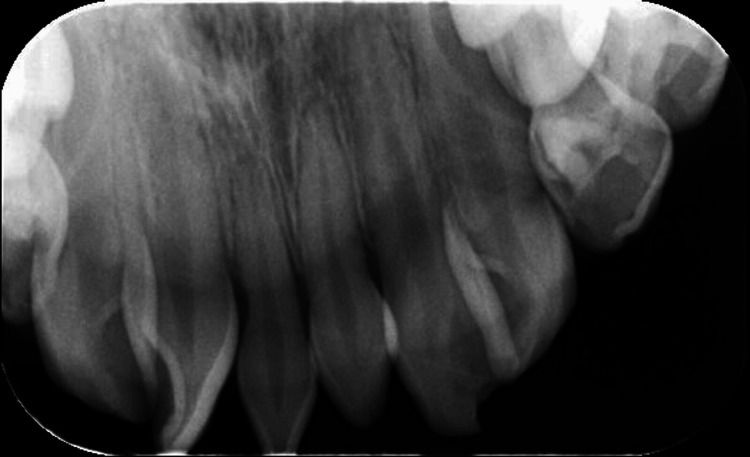
Periapical radiograph of the anterior maxillary region

The patient's history included a dental trauma nine months earlier, when she fell at home and injured her upper left permanent central incisor (tooth 21) on the edge of a bed. Clinical examination showed an uncomplicated crown fracture, with the tooth remaining vital, stable, and non-mobile (Figure 1). Radiographic imaging revealed complete root formation without any signs of periapical pathology or root fracture (Figure 2).

Born on April 4, 2015, by spontaneous vaginal delivery, the patient experienced hypotonia and poor feeding in the neonatal period, requiring a 12-day stay in the neonatal intensive care unit. Genetic testing in July 2016 confirmed a diagnosis of chromosome 1p36DS. She has been followed by a multidisciplinary team since then. Neurological follow-up for global developmental delay continued until age six, with no reported seizures. She is currently under ophthalmological care for alternating esotropia, with planned strabismus surgery. Aside from these issues, she has no chronic medical conditions and is not on long-term medications. In July 2023, she was re-evaluated at her local hospital and referred to a tertiary hospital for specialized dental management, as well as to otolaryngology and genetics.

The patient’s oral health history indicated poor hygiene, with irregular toothbrushing (twice weekly) using fluoridated toothpaste and no flossing. Feeding history included breastfeeding for one month, followed by prolonged bottle-feeding until age three. Her diet featured frequent sugary snacks, mainly chocolate and chips, in addition to three main meals per day. This was her first dental visit.

Behaviorally, she was classified as Frankl IV, demonstrating a cooperative and positive attitude. Non-pharmacological behavior management strategies, including the tell-show-do technique, positive reinforcement, distraction, and rewards, were successfully used. Her growth parameters were within normal limits: height 129 cm and weight 26 kg, aligning with the 50th percentile for Saudi standards.

Clinical examination

**Figure 3:**
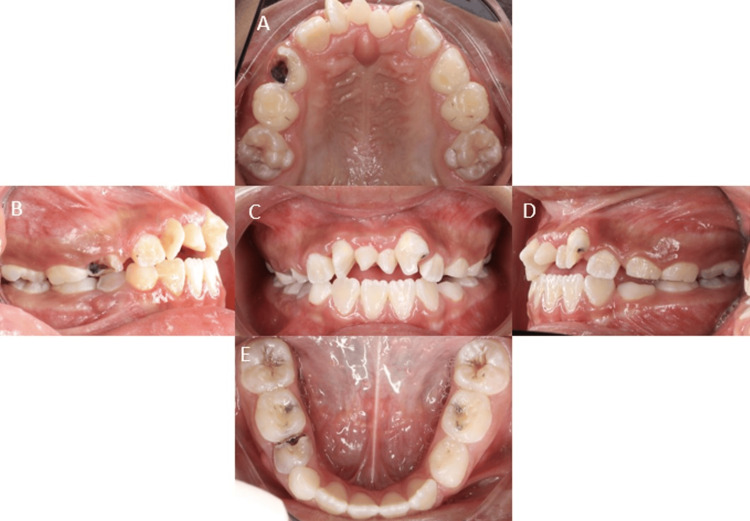
Intraoral photographs A: Upper occlusal view, B: Right buccal view, C: Frontal view, D: Left buccal view, E: Lower occlusal view

Extraoral examination showed a straight facial profile with mild asymmetry, a broad nasal bridge, and deviation of the nasal septum. The temporomandibular joint functioned normally, lips were competent at rest, and no cervical lymphadenopathy was noted. Intraorally, there was generalized plaque-induced gingivitis, physiological pigmentation, and a dentoalveolar abscess associated with tooth #84. Hard tissue assessment revealed multiple carious lesions, poor oral hygiene, and a plaque index score of 3 (Simplified Green and Vermilion Index). Other findings included dens invaginatus in teeth #12-22 and infraocclusion of tooth #74 (Figure 3). Occlusal analysis identified a bilateral Class II ½ molar relationship; the canine relationship could not be determined due to agenesis. The overjet was 0.5 mm, and the overbite was minimal. Both dental arches were U-shaped and symmetrical (Figure 3).

**Figure 4:**
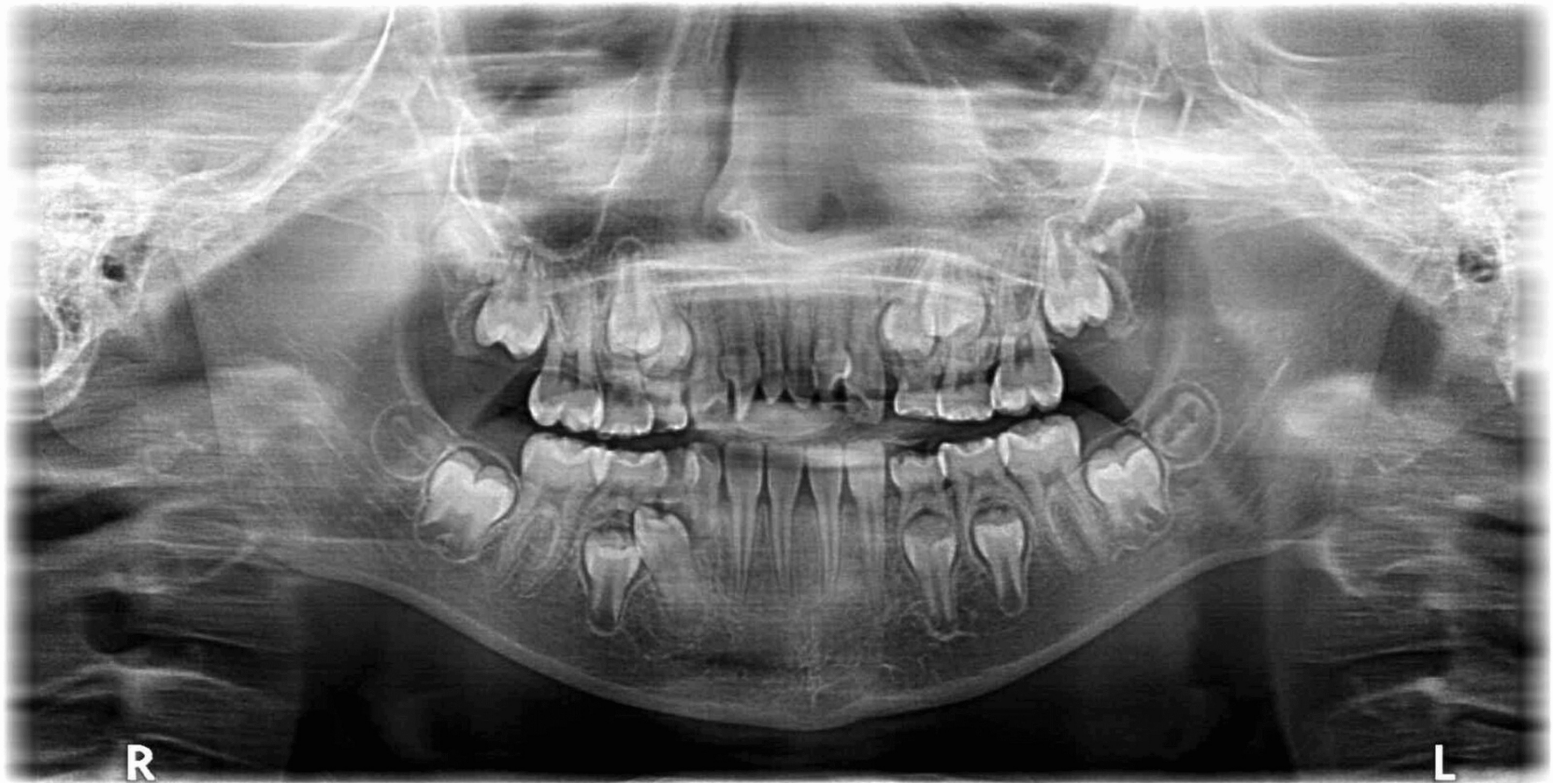
Panoramic radiograph of the patient

Panoramic radiography confirmed clinical findings, showing deviation of the nasal septum to the right, two supernumerary teeth in the anterior maxilla causing severe rotation of teeth #11 and #21, agenesis of all primary and permanent canines except for a retained mandibular right primary canine (#83), and ectopic eruption of the mandibular right first premolar (#44) (Figure 4).

Using the AAPD caries risk assessment tool, the patient was categorized as high risk due to inconsistent oral hygiene, frequent sugar consumption, multiple carious lesions, and limited professional fluoride exposure.

Treatment plan and clinical course

The main goals of treatment were to restore esthetics and psychosocial well-being, enhance oral hygiene and caries resistance, restore function, and address malalignment with interceptive orthodontics.

The preventive phase included reinforcing oral hygiene habits and dietary counseling. The patient and her family were instructed in twice-daily brushing with fluoridated toothpaste using the scrubbing technique, as well as daily flossing. Dietary advice focused on reducing the frequency of sugary snacks. Professional oral prophylaxis and topical fluoride varnish application were performed to help prevent caries.

During the restorative and surgical phase, all carious lesions were treated with tooth-colored restorative materials. The two mesiodens in the anterior maxilla were surgically extracted to relieve crowding, facilitate correction of rotated maxillary central incisors, and improve esthetics.

**Figure 5:**
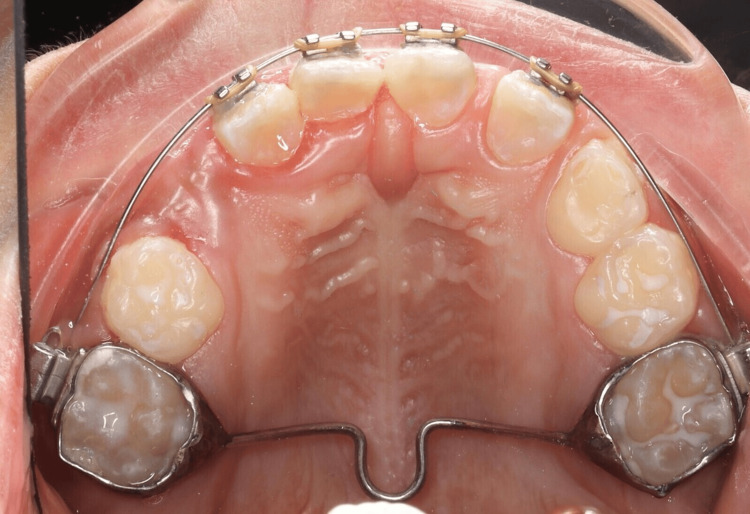
Intraoral photograph showing a cemented transpalatal arch (TPA) for anchorage control

**Figure 6:**
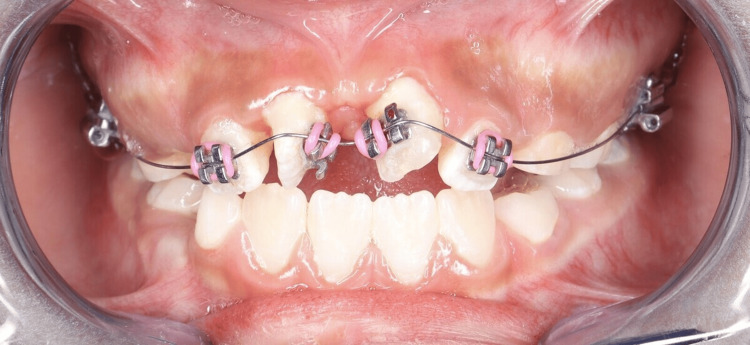
Intraoral photograph showing a bonded 2×4 fixed appliance with a 0.012 NiTi archwire used for alignment of the rotated maxillary central incisors (#11 and #21)

**Figure 7:**
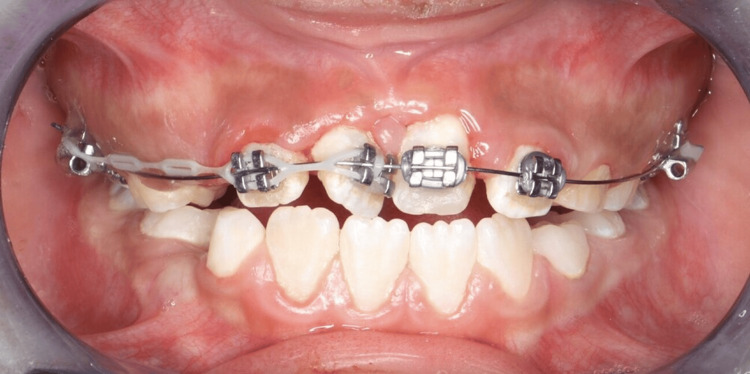
Intraoral photograph showing initial orthodontic alignment with 0.014 NiTi archwire after eight weeks, with a power chain extending from tooth #11 to the band on tooth #16 to aid in correction of severe rotation

**Figure 8:**
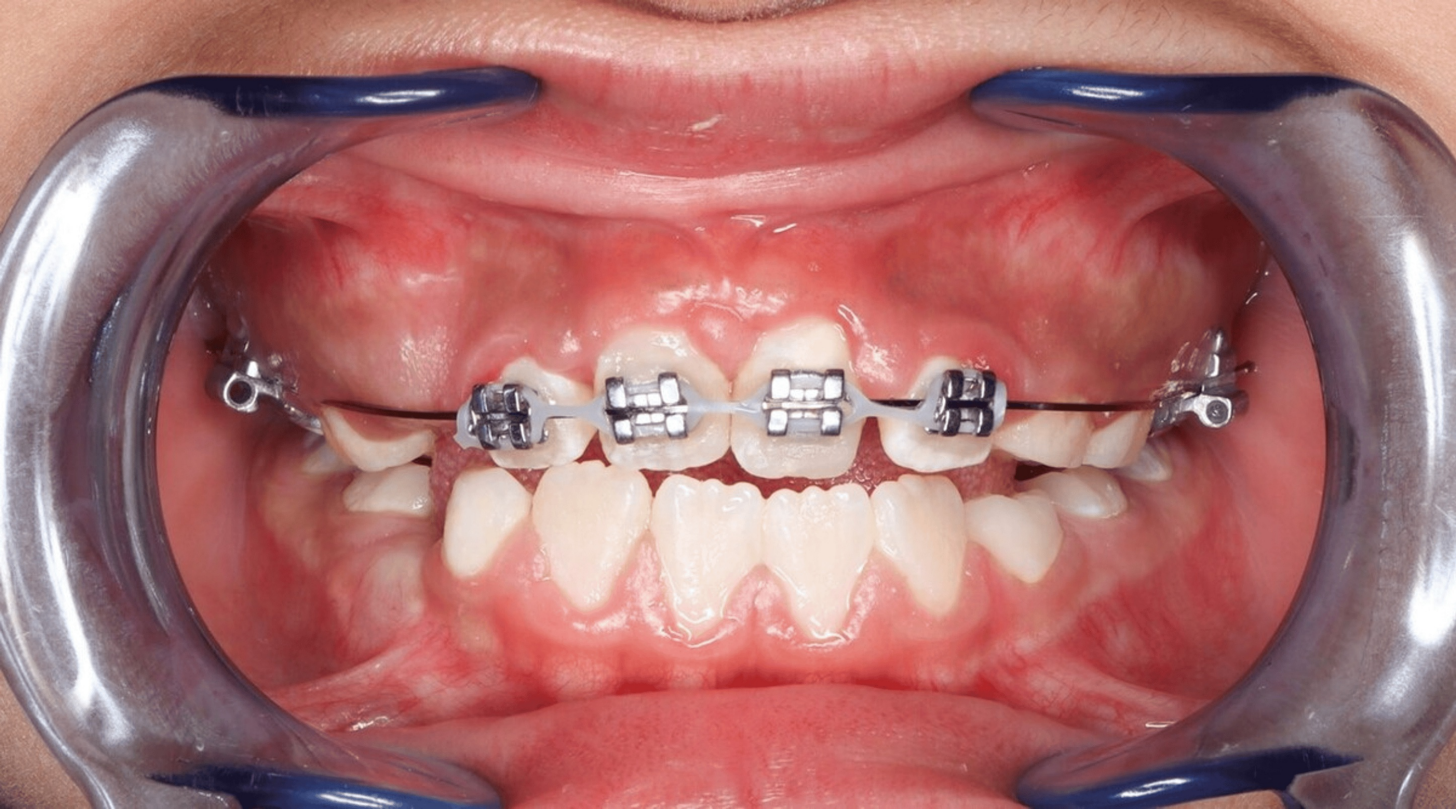
Intraoral photograph at 16 weeks showing a 0.016×0.022 stainless steel archwire with continued power chain activation to close residual spaces

**Figure 9:**
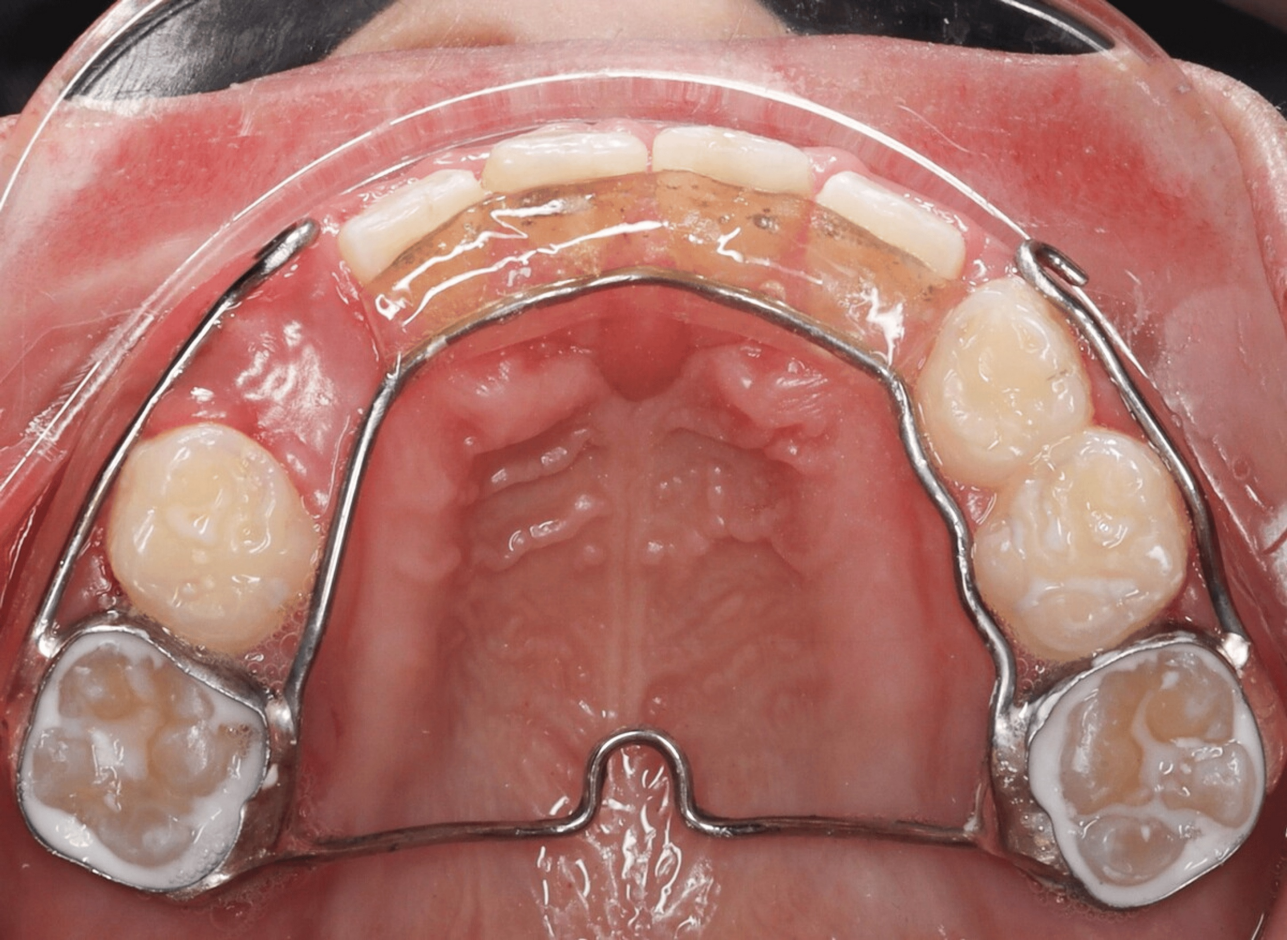
Intraoral photograph at 20 weeks showing the appliance modified to act as a retainer, with subsequent impressions obtained for a facemask-supported TPA to address the retrognathic maxilla and edge-to-edge incisal relationship TPA: Transpalatal arch

Orthodontic treatment followed. A transpalatal arch (TPA) was cemented to provide anchorage, and a 2×4 fixed appliance was bonded to align the rotated maxillary central incisors (#11 and #21) (Figures 5, 6). Initial alignment was achieved using a 0.012 NiTi archwire, which was progressively upgraded to 0.014 NiTi at eight weeks and 0.016 NiTi at 12 weeks. A power chain from tooth #11 to the band on tooth #16 was used to correct severe rotations (Figure 7). At 16 weeks, the archwire was upgraded to 0.016×0.022 stainless steel, with continued power chain activation to close residual spaces (Figure 8). At the 20-week visit, the appliance was modified to function as a retainer, and impressions were taken for a facemask-supported TPA to address a retrognathic maxilla and edge-to-edge incisal relationship (Figure 9). At 24 weeks, the facemask appliance was delivered with comprehensive instructions.

**Figure 10:**
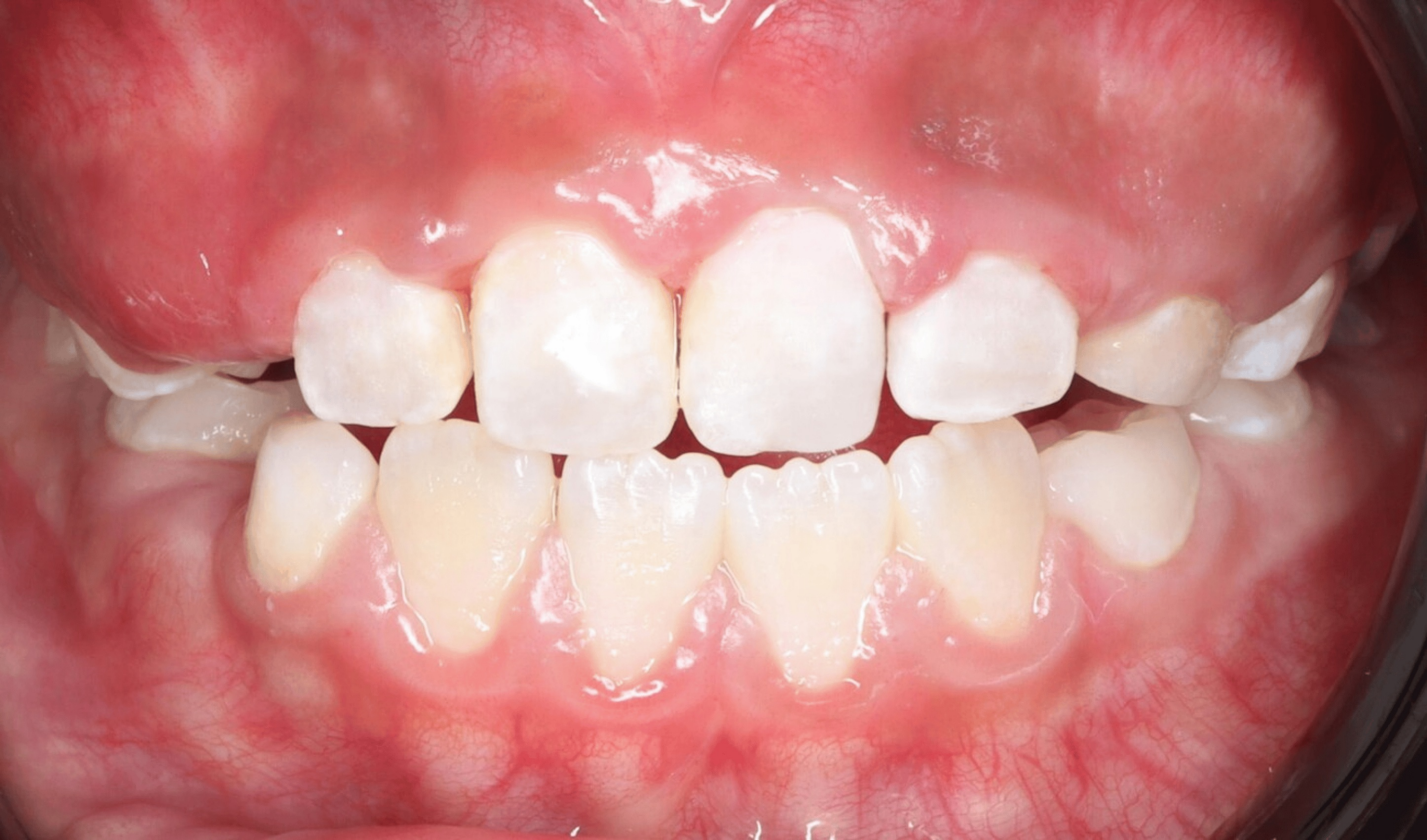
Intraoral frontal view after treatment showing improved alignment of the maxillary central incisors and establishment of a more esthetic anterior dental appearance

Recall appointments were scheduled every three months to match the patient’s high caries risk. Each visit included oral hygiene reinforcement, evaluation of restorations, application of topical fluoride, and orthodontic adjustments. The eruption sequence and occlusal development were closely monitored (Figure 10).

## Discussion

Chromosome 1p36DS is the most prevalent terminal chromosomal deletion, with a prevalence of 1 in 5,000 to 1 in 10,000 live births, accounting for about 0.5-1.2% of syndromic intellectual disability cases [2,6]. Its clinical presentation is highly variable, encompassing developmental delay, hypotonia, seizures, craniofacial dysmorphism, congenital heart disease, and varying degrees of intellectual disability [6,8]. This heterogeneity is largely attributable to differences in the size and content of the deletions, as well as structural rearrangements, leading to different levels of gene loss across the 1p36 region [8,9]. While systemic and neurological features have been well studied, dental findings have only recently gained recognition as potential diagnostic indicators [10].

Recent studies underscore that dental anomalies are an underappreciated but significant aspect of 1p36DS. Reports of dental agenesis and morphological changes like peg-shaped incisors and enamel hypomineralization in individuals with germline mosaicism for 1p36 deletion suggest that dental findings may be part of the syndrome’s broader phenotypic spectrum [2]. The case presented here is unique in that it shows both agenesis of all primary and permanent canines and the presence of supernumerary maxillary incisors (mesiodens), a combination not previously described. The presence of both agenesis and supernumerary teeth in a single patient points to a complex disruption of odontogenic signaling pathways, potentially involving genes in the deleted region such as SKI, GABRD, and MMP23B, which play roles in craniofacial development and morphogenesis [8]. These findings support the notion that the 1p36 locus is important for dental tissue patterning, and alterations in gene dosage here may affect epithelial-mesenchymal interactions critical to tooth initiation and morphogenesis.

Treating patients with 1p36DS necessitates a multidisciplinary approach due to the involvement of multiple systems. Craniofacial anomalies, muscular hypotonia, and cardiac dysfunction can complicate dental treatment, especially under sedation or general anesthesia [1]. Previous research has highlighted the need for thorough preoperative assessment and airway management preparedness, given the increased risk of difficult intubation and aspiration [1]. In this case, a tailored approach combining preventive, restorative, and interceptive orthodontic interventions led to improved function and esthetics. Early extraction of supernumerary teeth, correction of crowding, and alignment of rotated incisors contributed to restored occlusal harmony and greater patient confidence. This highlights the essential role of pediatric dentists not only in oral rehabilitation but in supporting psychosocial well-being and overall quality of life.

Clinically, pediatric dentists are often the first to detect syndromic dental anomalies. Routine panoramic imaging and careful attention to unusual combinations of dental anomalies, such as agenesis with supernumerary teeth or enamel defects, should prompt consideration of underlying chromosomal deletions and timely genetic referral. Preventive measures are especially important due to the high caries risk in these patients, stemming from poor oral hygiene, dietary factors, and neuromotor deficits [2]. Customized oral health programs, fluoride treatments, and periodic recall visits are vital to maintaining oral health stability. Furthermore, collaboration with specialists in genetics, cardiology, and anesthesiology is key to providing safe and comprehensive care, particularly for surgical or orthodontic needs.

This report adds to the expanding phenotypic spectrum of 1p36DS by documenting a previously unreported dental pattern. Recognition of such presentations has significant diagnostic value, enabling earlier referral for genetic testing. Pediatric dentists are instrumental in identifying syndromic clues, managing complex oral anomalies, and coordinating interdisciplinary care. Early diagnosis and management can improve oral function, esthetics, and psychosocial adaptation, illustrating the broader impact of dentistry on the holistic management of patients with rare genetic disorders.

## Conclusions

This case broadens the phenotypic spectrum of 1p36DS by presenting a previously unreported dental anomaly: the coexistence of supernumerary maxillary incisors and agenesis of both primary and permanent canines. These findings highlight the importance of comprehensive dental assessment as part of the multidisciplinary evaluation of affected individuals. Early identification of unusual dental patterns can facilitate timely genetic referral, support individualized treatment planning, and improve both functional and psychosocial outcomes. Pediatric dentists are crucial for recognizing syndromic indicators and coordinating interdisciplinary care, ensuring holistic management for children with rare chromosomal disorders.
